# APPLYING EVIDENCE-BASED PRACTICES TO INTERGENERATIONAL TECHNOLOGY-SUPPORTED LEARNING OPTIMIZES MUTUAL BENEFIT

**DOI:** 10.1093/geroni/igad104.1165

**Published:** 2023-12-21

**Authors:** Shannon Jarrott

**Affiliations:** The Ohio State University, Columbus, Ohio, United States

## Abstract

Numerous intergenerational programs support varied goals using technology. Technological competence may be the desired outcome or the means for achieving outcomes in such programs. For example, Cyber Seniors operates in many settings with digital mentors providing technology training to more than 11,000 older adults. This and other technology-supported intergenerational programs have been delivered in-person and remotely with considerable success. Now that researchers have demonstrated feasibility and acceptability of intergenerational technology-supported learning, they may want to address the potential for such programs to be one sided. In the typical scenario, the youth bestows their technological expertise upon the older learner, but the pair can mentor each other to achieve additional goals. Application of evidence-based intergenerational strategies can optimize mutual benefit of technology-supported learning programs. In this paper, specific practices are illustrated with international examples from diverse intergenerational programs that incorporate technology. The author’s and others’ research linking practice with outcomes reveals that humanistic practice strategies (e.g., supporting equal group status in the contact setting) ensure that youth and older adults build relationships as well as technical skills while they learn from each other.

